# Feasibility and acceptability of remotely monitoring spirometry and pulse oximetry as part of interstitial lung disease clinical care: a single arm observational study

**DOI:** 10.1186/s12931-024-02787-1

**Published:** 2024-04-15

**Authors:** Sarah Barth, Colin Edwards, Gauri Saini, Yussef Haider, Nicholas Paul Williams, Will Storrar, Gisli Jenkins, Iain Stewart, Melissa Wickremasinghe

**Affiliations:** 1 Imperial College Healthcare NHS Trust, ILD Service, Mint Wing, St Mary?s Hospital, Praed Street, London, W2 1NY UK; 2 PatientMpower Ltd, Dublin, Ireland; 3 Nottingham University Hospitals NHS Trust, Nottingham, UK; 4 Lancashire Teaching Hospitals NHS Trust, Preston, UK; 5 Hampshire Hospitals NHS Foundation Trust, Winchester, UK; 6 Hampshire Hospitals NHS Foundation Trust, Basingstoke, UK; 7 National Heart & Lung Institute, Imperial College London, London, UK

**Keywords:** Interstitial lung disease, Physiological monitoring, eHealth

## Abstract

**Background:**

Remote monitoring of patient-recorded spirometry and pulse oximetry offers an alternative approach to traditional hospital-based monitoring of interstitial lung disease (ILD). Remote spirometry has been observed to reasonably reflect clinic spirometry in participants with ILD but remote monitoring has not been widely incorporated into clinical practice. We assessed the feasibility of remotely monitoring patients within a clinical ILD service.

**Methods:**

Prospective, single-arm, open-label observational multi-centre study (NCT04850521). Inclusion criteria included ILD diagnosis, age ≥ 18 years, FVC ≥ 50% predicted. 60 participants were asked to record a single spirometry and oximetry measurement at least once daily, monitored weekly by their local clinical team. Feasibility was defined as ≥ 68% of participants with ≥ 70% adherence to study measurements and recording measurements ≥ 3 times/week throughout.

**Results:**

A total of 60 participants were included in the analysis. 42/60 (70%) were male; mean age 67.8 years (± 11.2); 34/60 (56.7%) had idiopathic pulmonary fibrosis (IPF), Median ILD-GAP score was 3 (IQR 1–4.75). Spirometry adherence was achieved for ≥ 70% of study days in 46/60 participants (77%) and pulse oximetry adherence in 50/60 participants (83%). Recording ≥ 3 times/week every week was provided for spirometry in 41/60 participants (68%) and pulse oximetry in 43/60 participants (72%). Mean difference between recent clinic and baseline home spirometry was 0.31 L (± 0.72). 85.7% (IQR 63.9–92.6%) home spirometry attempts/patient were acceptable or usable according to ERS/ATS spirometry criteria. Positive correlation was observed between ILD-GAP score and adherence to spirometry and oximetry (rho 0.24 and 0.38 respectively). Adherence of weekly monitoring by clinical teams was 80.95% (IQR 64.19–95.79). All participants who responded to an experience questionnaire (*n* = 33) found remote measurements easy to perform and 75% wished to continue monitoring their spirometry at the conclusion of the study.

**Conclusion:**

Feasibility of remote monitoring within an ILD clinical service was demonstrated over 3 months for both daily home spirometry and pulse oximetry of patients. Remote monitoring may be more acceptable to participants who are older or have more advanced disease.

**Trial Registration:**

clinicaltrials.gov NCT04850521 registered 20^th^ April 2021

**Supplementary Information:**

The online version contains supplementary material available at 10.1186/s12931-024-02787-1.

## Introduction

Interstitial lung disease (ILD) is used to describe a group of diseases with differing aetiology that share clinical, radiological and histopathological features of progressive fibrosis or inflammation of the lung parenchyma. Both inflammation and fibrosis of the lungs affect oxygen exchange across the alveolar membrane and so cause progressive hypoxaemia and dyspnoea and can also lead to right sided heart failure. Idiopathic pulmonary fibrosis (IPF) is the most common idiopathic ILD [1].

Usual clinical monitoring of patients with IPF is outlined in internationally agreed guidelines [2, 3] which recommends clinical review every 4–6 months with pulse oximetry and spirometry assessment to assess respiratory physiology. There are no formal guidelines for the monitoring of patients with other forms of ILD but similarly they depend on serial lung function measurement to guide and measure the response to therapy [4]. Physiological decline in forced vital capacity (FVC) of > 10% over 1 year has been shown to be associated with worse clinical outcomes regardless of radiological or histopathological diagnosis [5]. Antifibrotic therapy such as nintedanib or pirfenidone has been shown to slow the physiological decline associated with both IPF [6, 7] and progressive fibrosis in ILD [8].

Remote patient monitoring has the potential to support consultations through the provision of real time clinical measurements that can contribute to shared decision making, earlier identification of exacerbations and monitoring of treatment responses. Suggested benefits for patients include reduced travel to hospital settings and improved accessibility, more awareness of disease trajectory, earlier identification of deterioration [9], and increased confidence at self-management [10, 11].

The Covid-19 pandemic has highlighted the need for virtual care solutions to be integrated into clinic systems as part of pandemic preparedness [12], although limited evidence to support rapid uptake has been criticised [13].

There is limited experience of remote pulse oximetry in the care of patients with ILD although previous surveys have shown that this is the test that most clinicians feel would be most useful to monitor [14]. It has been proposed to use remote pulse oximetry as a method to determine need for domiciliary oxygen therapy [15] and it has been demonstrated that patients with chronic lung disease find home oximetry useful [16]. Retrospective studies of patients with Covid-19 have shown that remote pulse oximetry monitoring is safe [17, 18].

Previous studies have demonstrated that remotely recorded spirometry measurements correlate well with hospital-based spirometry measurements for both participants with IPF [9] and non-IPF ILDs [19], whilst feasibility in asking participants to record remote spirometry on a daily or weekly basis has been demonstrated separately from clinical service [20–23]. Prospective studies of remote monitoring of spirometry in IPF have shown a positive impact on participants’ psychological measures, as well as participant satisfaction with the ease and utility of remote monitoring programmes [11, 24].

Clinicians and health services have an urgent need to understand how remotely captured data should be managed, used and integrated within the clinical setting and to identify which participants would appreciate remote monitoring and benefit the most.

This study aimed to assess the feasibility of daily remote monitoring of spirometry and pulse oximetry for the purpose of clinical review to monitor their disease status to support their ongoing clinical care.

## Methods

### Study Population

Participants with an established diagnosis of fibrotic ILD were prospectively recruited from 4 respiratory clinics in England. Inclusion criteria were age ≥ 18 years, FVC ≥ 50% predicted, access to a smartphone or tablet device with internet connectivity, access and use of an email address, fluency in English language and willingness to comply with study procedures. Exclusion criteria included a diagnosis of cognitive impairment, other serious medical conditions which may cause respiratory distress and involvement in another concurrent research project.

### Study Design

Participants were enrolled in a single arm observational study lasting 13 weeks. At baseline participants were provided with access to an application (pMp, patientMpower Ltd) to download onto their smartphone/tablet device and a personal handheld spirometer (Spirobank Smart, MIR) and pulse oximeter (Nonin 3230) both of which linked to the pMp app via Bluetooth. Participants were supported to link the devices to the app via Bluetooth and given instruction on how to provide measurements. At baseline the participants’ age, sex, smoking history, diagnosis and most recent clinic spirometry were recorded.

Participants were asked to record a single spirometry and oximetry measurement at least once daily for 91 days. All patient-recorded data was immediately available to view by their clinical centres via a secure browser-based portal. Clinical centres were asked to monitor these data at least once per week and were asked to respond to abnormal measurements. Weekly monitoring was requested as a pragmatic interval to monitor changes in lung function. As the study measured adherence as a measure of feasibility, clinical teams were asked not to contact patients regarding adherence to study measurements. Clinical interactions during the study period were recorded.

Participants were asked to complete the EQ-5D-5L questionnaire [25] and Patient Activation Measure® (PAM®) [26, 27] at baseline and conclusion of the 91 days and an experience survey at the conclusion of the study. The EQ-5D-5L was used to assess health related quality of life with higher scores reflecting better quality of life. Estimated MID for the EQ-5D-5L is 0.095 for index score and 9.7 for the visual analogue score (VAS) [28]. The PAM questionnaire was used to measure patient engagement in healthcare with higher scores reflecting patients who are more engaged. MCID for PAM is 4 points [27, 29].

The severity of the patients’ ILD was assessed using the ILD-GAP score [30] which estimates mortality throughout the spectrum of the ILDs and has been used as a proxy for severity of disease.

This study was designed to assess the feasibility of remote digital monitoring within a clinical service provision by assessing whether the use of home monitoring devices by participants was sustained. The primary endpoints selected to reflect this were (A) number of participants with 70% adherence to daily study measurements (over 91 days); (B) number of participants who recorded ≥ 3 measurements per week through the study duration to reflect persistence throughout the study.

### Sample size

According to defined primary endpoints, adherence to daily spirometry in the INJUSTIS study interim analysis of mixed fibrotic ILD [19] indicated that 68% participants (52/76) recorded daily home spirometry on ≥ 70% study days (observation period 105 days). The sample size (*n* = 60) was selected pragmatically with the expectation that ≥ 41 participants would complete the feasibility endpoint.

### Statistical analysis

Adherence was calculated as the number of days with recorded measurements/total duration of study (91 days) expressed as a percentage.

Correlation between home and hospital spirometry was assessed using intraclass correlation coefficients in a two-way random effects model. Bland-Altman plots assessed the difference between the most recent clinical spirometry record prior to recruitment and the highest value of the first day of independent home spirometry to determine the 95% limits of agreement.

Spirometry data was retrospectively analysed using artificial intelligence software (ArtiQ.QC) to validate spirometry [31] against ERS/ATS 2019 criteria [32, 33] and was either classed as acceptable/usable or rejected. The coefficient of variation in home spirometry measures (calculated as 100*standard deviation/mean) was assessed for each month of the study with and without spirometry acceptability criteria applied. Sensitivity analysis was also performed on primary endpoints based on acceptability criteria.

Correlations with continuous variables were assessed with Pearson correlation, or Spearman for ordinal variables.

Change in PAM score was assessed using Wilcoxon signed rank test, differences across ethnicity and ILD Subtype with adherence were analysed using Kruskal Wallis test. The demographics of responders to the patient experience survey were analysed using Mann Whitney U testing and chi squared testing. Significance was defined as *p* < 0.05, all tests were performed with Stata.

### Approvals

The study received ethical approval from the East Midlands-Derby Research Ethics Committee and the Health Research Authority. The study was conducted in accordance with the ethical principles of the Declaration of Helsinki. All participants provided informed consent.

## Results

Sixty-two participants gave informed consent to enter the study. The analysis includes only the participants who downloaded the app and provided at least one spirometry and one oximetry measurement (*N* = 60). Participants were majority male (70%), had an IPF diagnosis (57%), a mean age of 67.75 (SD 11.19) and a mean percent predicted FVC of 84.30 (SD 19.84) (Table 1). Median interval between most recent clinic spirometry and start of home spirometry was 162 days (IQR 89–252 days).

**Table 1:** Study demographics

		N (%)/mean (±SD)
Total number of participants		60
Age		67.78 (± 11.13)
Gender	Male Female	42 (70) 18 (30)
Ethnicity	White Asian Black/Other/Not Reported	49 (82) 6 (10) 5 (12)
ILD diagnosis	IPF Chronic Hypersensitivity Pneumonitis (CHP) Rheumatoid Arthritis Associated ILD (RA-ILD) Connective Tissue Disease Associated ILD (CTD-ILD) Other (unclassifiable ILD, sarcoidosis, idiopathic nonspecific interstitial pneumonia (NSIP), asbestosis, familial ILD)	34 (57) 5 (80) 6 (10) 7 (11) 8 (13)
Smoking status	Ever smoker (ex or current) Never smoker	43 (72) 17 (28)
FVC (L)		3.09 (± 1.12)
FVC (% predicted)		84.33 (± 19.81)
TLCO (% predicted)		53.88 (± 18.20)
ILD-GAP score [median]		3 (IQR 1–4.75)

The study achieved both its primary endpoints of ≥ 68% of participants recording daily home measurements on ≥ 70% of days and ≥ 3 times/week during the observation period for both spirometry and pulse oximetry (Table 2).

**Table 2:** Feasibility of remote recording of spirometry and pulse oximetry by participants with ILD

	Spirometry	Pulse oximetry
Participants recording ≥ 3 days/week every week [n (%)]	41 (68)	43 (72)
Participants recording ≥ 70% of study days [n (%)]	46 (77)	50 (83)

Median adherence throughout the study was 91.2% (IQR 72.2–95.6) for spirometry and 91.2% (IQR 74.4–95.6) for pulse oximetry. The proportion of participants recording at least one measurement at least once per week was 45/60 (75%) for spirometry and 47/60 (78%) for pulse oximetry. The frequency of home spirometry showed high levels of adherence to remotely recording measurements by the majority of patients (Additional Fig. 1).

There was one reported adverse event during the study in which a patient experienced a fainting episode during a forced expiratory manoeuvre.

Adherence to weekly review by clinical teams was 80.95% (IQR 64.19–95.79). It was not recorded how these impacted on clinical interactions with the patients.

Two participants were admitted to hospital during the observation period. These events were not precipitated by the recording of study measurements. Two participants were advised to discontinue remote spirometry by their ILD clinicians during the study– one was advised to discontinue due to a medical event in which spirometry was contraindicated and the other due to syncopal symptoms during spirometry.

**Fig. 1:**
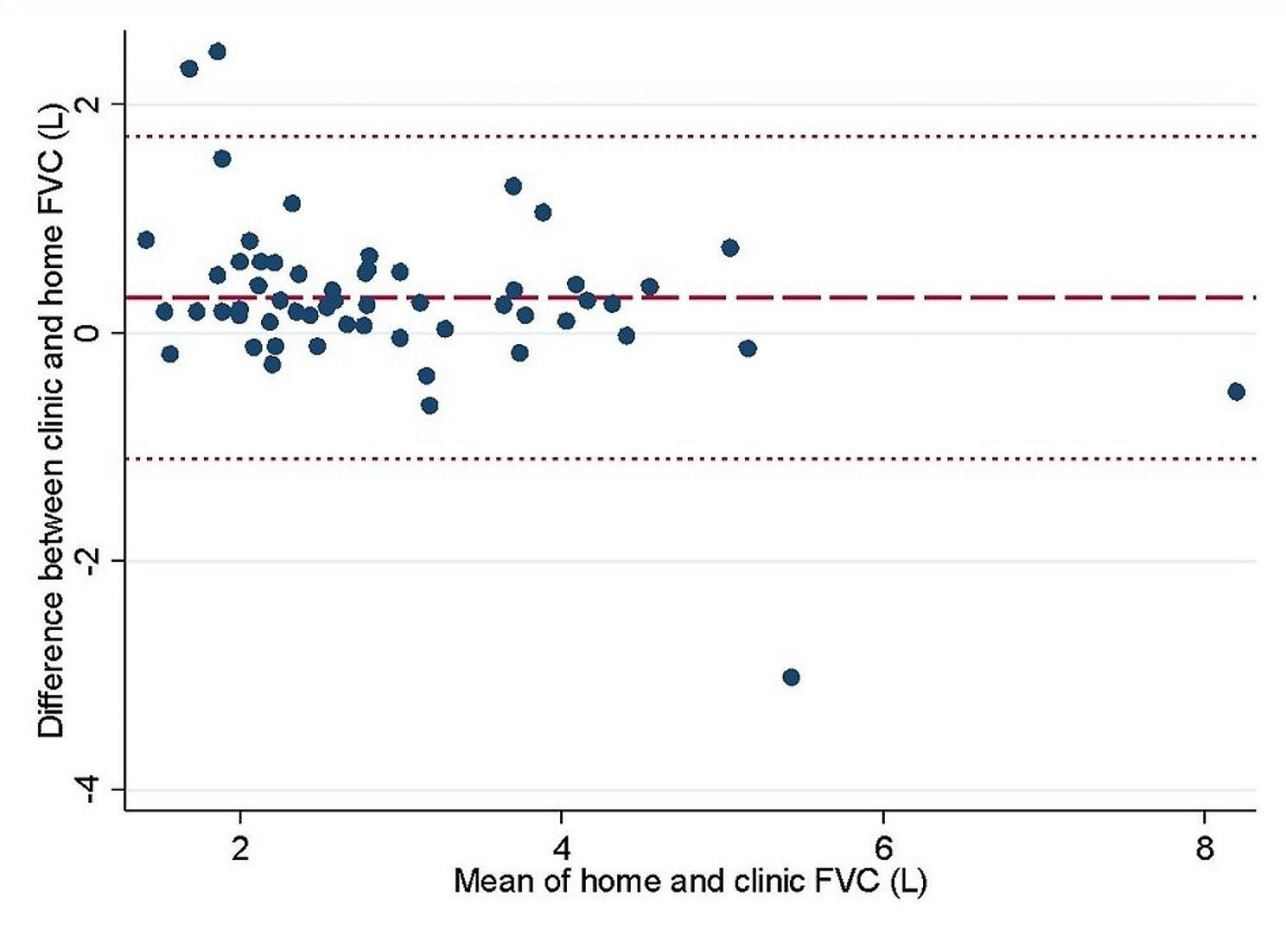
Bland Altman analysis of home spirometry vs. most recent clinic recorded spirometry; Home spirometry measurement was the highest spirometry value recorded on the first independent day of spirometry during the study to allow for multiple attempts due to coughing. Clinic spirometry was the most recently recorded spirometry either in the lung function department or performed under supervision of a clinician. Dotted lines indicate 95% limits of agreement

Hospital measured spirometry records were obtained in 57/60 (95%) participants a median of 162 days prior to starting the study (IQR 89–252 days). High correlation was observed between most recent hospital recorded spirometry and remotely recorded spirometry at start of study (rho 0.83 *p* < 0.001). The 95% agreement limits of home recorded spirometry values with most recent clinic recorded values were − 1.1 L and 1.72 L (Fig. 1). Mean difference between recent clinic and first independent home spirometry was 0.31 L (± 0.72) with clinic spirometry tending to be higher than home recordings.

The within-subject coefficient of variation was 16.77% for all spirometry attempts in month 1 (Table 3). Across the entire study, 74.81% of all spirometry attempts were categorised as either acceptable or usable with a median 85.7% (IQR 63.9–92.6%) spirometry attempts per participant. The within subject coefficient of variation was 9.1% for spirometry attempts in month 1 when restricted to those categorised as acceptable or usable. Within-subject variability was lower in later months. Restriction of primary endpoints based on acceptability criteria, 45% of participants achieved 70% spirometry adherence and 43% of participants recorded spirometry ≥ 3 days per week.

**Table 3:** Coefficient of variation throughout study in all spirometry blows and in those validated

	Day 1-30	Day 31-60	Day 61-90
Within subject coefficient of variation: All spirometry attempts included	16.77% (± 12.2)	10.20% (± 10.55)	11.33% (± 13.53)
Within subject coefficient of variation: Only spirometry attempts classed as usable or acceptable included	9.10% (± 6.24)	8.15% (± 8.18)	7.44% (± 11.24)

**Fig. 2:**
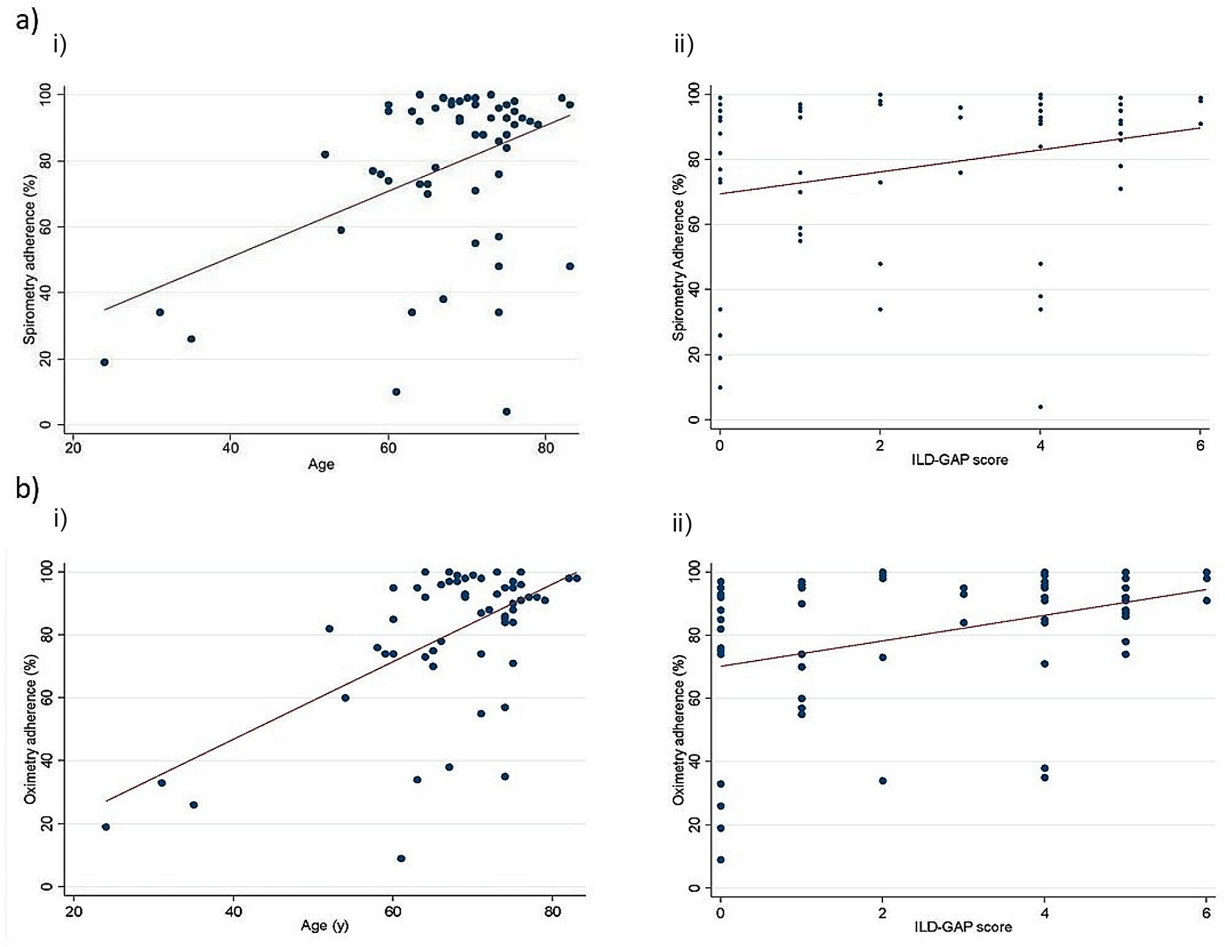
Correlation of adherence to daily spirometry (a) and pulse oximetry (b) with (i) age and (ii) ILD GAP score. Graph demonstrating increased adherence to study spirometry measurements in patients who are (i) older and (ii) with more severe disease

Correlation between age and adherence to spirometry was weak and did not reach significance (rho 0.22; p *=* 0.09), although positive correlation was observed in adherence to remote oximetry (rho 0.38; *p* = 0.002). Positive correlation was observed between the ILD-GAP score and adherence to both spirometry (rho 0.29, *p* = 0.02) and oximetry (rho 0.38 *p* = 0.003). A significant difference was also noted between ethnicity and adherence to study measurements (p 0.02 and 0.04 for spirometry and oximetry respectively). No significant correlation was observed between any other variable (Table 4; Fig. 2).

**Table 4:** Assessment of relationship between demographic data and adherence to study measurements

			Spirometry adherence		Oximetry Adherence	
			Correlation	p	Correlation	p
Age			0.22	0.09	0.38	0.002
Baseline FVC			-0.17	0.21	-0.13	0.34
Baseline FVC (% pred)			-0.05	0.71	-0.11	0.42
Baseline TLCO			-0.12	0.36	-0.19	0.14
ILD-GAP score			0.2903	0.02	0.38	0.003
ILD-GAP stage			0.24	0.056	0.300	0.02
Baseline EQ-5D-5 L index score (*n* = 46)			0.18	0.23	0.19	0.21
Baseline vas			0.446	0.03	0.4632	0.03
		n	Median (IQR)	*P*	Median (IQR)	*P*
Gender^†^	Male	42	90 (73–95)	0.86	91 (74–97)	0.35
	Female	18	88 (72–95)		86.5 (75.25–94.5)	
Ethnicity^†^	White	49	93 (74–97)	0.02	92 (76–97)	0.04
	Asian	6	89.5 (88–91.75)		89.5 (87.25–91.75)	
	Black/Other / Not reported	5	34 (19–38)		38 (33–71)	
IPF vs. non-IPF^†^	IPF	34	92 (77.5-97.25)	0.08	92 (82.5–98)	0.07
	Non IPF -ILD	26	79.5 (53.25–95.25		85 (59.25–95.25)	
ILD subtype^†^	IPF	34	80.5 (43.25–93.75)	0.046	92 (82.5–98)	0.24
	CTD-ILD	7	95(82–97)		95 (82–97)	
	CHP	5	92(58–95)		92 (58.5–95.5)	
	RA-ILD	6	51.5 (40.75–70.75)		72.5 (46-88.25)	
	Other	8	85 (44-96.5)		80.5 (43.25–93.75)	

Correlations measured using Spearman’s rank correlation. ^†^Difference in adherence in gender, ethnicity and ILD subtype measured using Kruskal-Wallis analysis

Participants were asked to complete EQ-5D-5L and PAM questionnaires at beginning and end of the study. There was variable adherence to this so the numbers of responses are summarised in the tables to reflect the variable response rates.

A total 26/60 (43%) participants completed both a baseline and end of study EQ-5D-5L and there was no significant change in the EQ-5D-5L index value over the course of the study (median change − 0.02 IQR − 0.6–0.0, *p* = 0.077) but a significant decline was noted in the visual analogue score (VAS) (median change − 6 *p* = 0.017). (Table 5).

**Table 5:** Results of EQ-5D-5L questionnaire

	Start of study		End of study		Change over study		
	n	Median (IQR)	n	Median (IQR)	n	Median change in value (IQR)	p
EQ-5D-5L Index score	46	0.86 (IQR 0.67–0.95)	22	0.819 (IQR 0.745–0.988)	22	−0.02 (IQR − 0.6 – +0.0)	0.077
EQ-5D-5L Visual Analogue Score	48	80 (IQR 60–90)	22	70 (IQR 61.25–88.75)	22	-6.2 (IQR − 10.75 – +1.5)	0.0165*

All completed EQ-5D-5L questionnaires are summarised here. Index values which could not be calculated due to missing values were excluded (*n* = 2). Only participants who provided both beginning and end of study questionnaires were included in the analysis of change over the experiment results

A total 23/60 (38%) participants completed the PAM-13 at beginning and end of study. Median change in score between beginning and end was − 4.7 (IQR − 13.35 to + 2.4; *p* = 0.06) and median change in PAM level was 0 (IQR − 0.5 to 0.0, p *=* 0.35) (Table 6).

**Table 6:** Results of PAM-13 score

	Number of participants	PAM-13 Score Median (IQR)	PAM-13 Level Median (IQR)
Start of study	39	60.6 (33.5–73.95)	3 (3–3.5)
End of study	34	64.3 (55.6–72.5)	3 (3–4)
Change over study	23	−4.7 (IQR − 13.35 – +2.4)	0 (IQR − 0.5- 0)
p-value		0.06	0.35

A weak positive correlation was observed between first recorded PAM level and adherence to spirometry (rho 0.318, *p* = 0.024), but not oximetry (rho 0.26, *p* = 0.06) (Table 7).

Worsening HRQoL score and adherence to study measurements did not reach statistical significance. (Spirometry rho − 0.25 *p* 0.26 oximetry rho − 0.19 *p* 0.39) (Table 7).

**Table 7:** Correlation between questionnaire scores and adherence to study measurements

		Spirometry		Oximetry	
	Number of participants	Correlation	p-value	Correlation	p-value
EQ-5D-5L index score (baseline)	46	0.18	0.23	0.19	0.21
EQ-5D-5L VAS (baseline)	48	0.20	0.17	0.1953	0.18
Change in EQ-5D5L index score over the study duration	22	-0.25	0.26	-0.19	0.386
Change in Eq. 5D5L VAS score over the study duration	22	-0.249	0.26	-0.294	0.18
PAM-13 (first available score)	50	0.318	0.024*	0.26	0.06

**Fig. 3:**
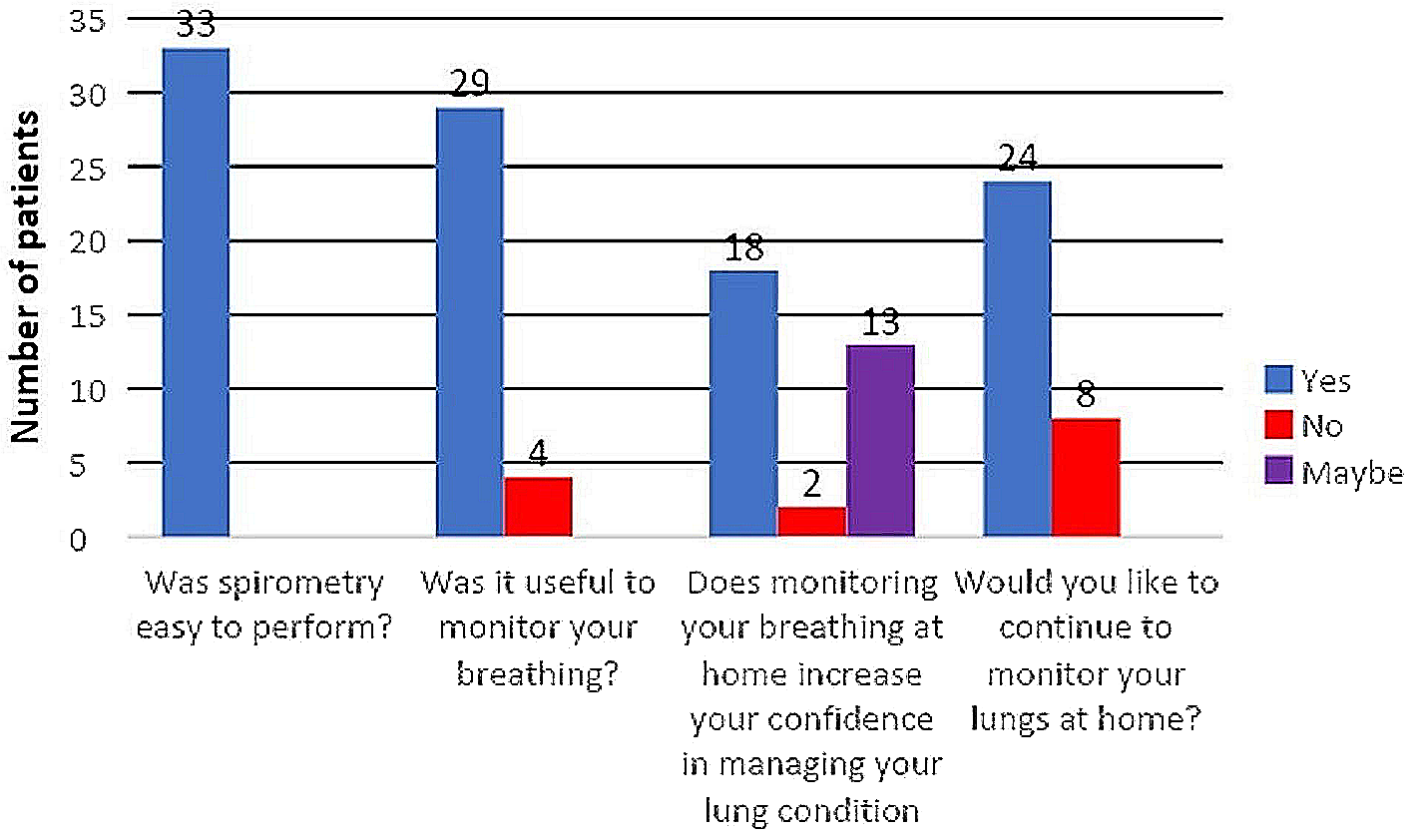
Results of an experience survey assessing acceptability of remote monitoring to participants; 33 participants (55%) responded at the conclusion of the study. One participant did not respond to the question about continuing remote monitoring

A total 33/60 (55%) participants responded to an opinion questionnaire at the conclusion of the study (Fig. 3). The participants who responded to the questionnaire had a higher adherence to study spirometry (93% vs. 81%, *p* = 0.02) and lower ILD-GAP score (2.5 vs. 3.0, *p* = 0.001). There was no significant difference observed between the participants who responded to the survey and those who did not in terms of age (*p* = 0.47), gender (*p* = 0.61), ILD subtype (*p* = 0.37) or baseline clinic recorded physiology (FVC, *p* = 0.56; TLCO, *p* = 0.77)). All respondents (33/33) stated home spirometry was easy to perform and 29 participants (88%) felt that it was useful to monitor their spirometry at home. 17/33 (51.5%) recommended daily frequency of measures, 12/33 (36.4%) weekly and the remainder suggested infrequently or not at all (4/33, 12.1%). 24/32 participants (75%) wished to continue remote monitoring beyond the study end date – 1 participant did not respond to this question. There was no signal of a statistically significant effect of baseline age, PAM score or FVC on participants’ desire to continue home monitoring (Additional Table 1).

## Discussion

This study demonstrated feasibility of daily monitoring of spirometry and oximetry to participants with ILD as part of their clinical care over 3 months. No safety issues were observed and there were no unexpected adverse events. The study showed acceptability of remote monitoring to those participants who responded to a survey.

This is the first UK based study to address adherence and acceptability of remote monitoring to participants in complementing usual ILD clinical care rather than as a research tool; and to consider what aspects may affect acceptability of remote monitoring to participants within this context, observing that worsening disease severity may be associated with increased acceptability of remote monitoring in clinical care.

High levels of adherence to remote digital monitoring of both spirometry and pulse oximetry in participants with ILD were observed during 3 months, both in terms of absolute adherence and persistence with weekly measurements, supporting clinical utility. Home recorded spirometry correlated closely with clinic spirometry although, as in other studies, there was underestimation of home spirometry compared with clinic spirometry [19]. The recent clinical spirometry records were an average of 162 days prior to study baseline. This may account for the negative mean difference and wide limits of agreement from − 1.19 L to 0.58 L between a participant’s average home spirometry and their clinical record. The length of the duration between home and clinic spirometry is likely to be due to backlog due to the COVID-19 pandemic.

Within-person variation of home spirometry was higher than that seen in other studies of remote spirometry in patients with ILD [9, 11, 20, 34]. This may be due to differences in calculation methods and the composition of disease subgroups. The coefficient of variation was lower with spirometry measurements which were categorised of acceptable quality. This demonstrates the need for real time quality assurance of home spirometry, although further improvement in this AI technology is needed given the wide variation still observed. Regular training of patients [35] has been proposed as a further method to improve the reliability of home monitoring in clinical practice. We observed lower levels of variability across subsequent study months suggesting a learning effect. The majority of home spirometry attempts/patient were acceptable according to ERS/ATS criteria which supports the feasibility and reliability of home spirometry.

Although the number of clinical interactions was recorded, information was not captured as to whether these were planned or unplanned or were attributable to clinical review of the remote spirometry and/or oximetry data. This will need to be further explored to understand the clinical utility of incorporating remote spirometry and oximetry into patient care.

This study has also looked at factors that may affect acceptability and therefore adherence to remote monitoring. Adherence to both spirometry and oximetry was positively correlated with ILD-GAP score, suggesting that participants with ILD who are older or have more severe disease may be more engaged with remote monitoring as a modality to monitor their lung disease. This contrasts with other studies which demonstrated earlier discontinuation from remote spirometry in older patients and those with increased dyspnoea [34]. One possibility is the inclusion of pulse oximetry which participants with more advanced disease may find more useful [16].

This current study was limited by relatively few participants having ILD-GAP stage III disease and lack of significant correlation of worsening HRQoL score with adherence to remote measures, highlighting the importance of investigating the relationship between adherence and disease severity in further detail.

A significant difference was also noted in adherence between patients of different ethnicities. The participant cohort was predominantly white/Caucasian with small numbers of participants of other ethnicities. However, this relationship will need to be further investigated in the future.

### Strengths and limitations

This study recruited participants between October 2021 and March 2022 during which the coronavirus pandemic was still affecting all aspects of how healthcare was delivered, with limited in person contacts and lung function availability. This impacted the availability of recent lung function data and wide variation of the observed duration between baseline clinic-based spirometry and start of the study. This may also have affected acceptability of home monitoring to participants, either by highlighting the potential for reduced in-person contact or alternatively by providing a method for monitoring their lung disease that was not affected by access to services. It is not clear what the long term impact of the pandemic will be on remote monitoring. Patient engagement with remote monitoring may be lower as patients wish to return to as much in person interactions as possible. However a recent study has shown that patients with long term chronic conditions wish to engage with technology to monitor their condition [36].

Secondary analyses were not corrected for multiple testing. This study used validated patient reported outcome measures as well as bespoke surveys to gather patient experience to further inform feasibility and acceptability. The analysis of PAM-13 was limited by the relatively small data set but suggests that participants who are more engaged in their healthcare were more likely to record remote clinical measurements. Fewer than 50% of participants responded to repeat PAM-13 and EQ-5D-5L questionnaires, limiting interpretation of change over time. The direction of effect between patient engagement and remote monitoring adherence is unknown and a longer time interval would be beneficial to understand longer term adherence as disease progresses. Similarly, a small but significant decline was noted in the participants HRQoL scores over the course of the study and it is unknown whether this is related to the remote monitoring intervention or the progressive nature of their underlying disease.

This study was limited by follow-up of 91 days and does not demonstrate whether remote monitoring in a clinical ILD service is feasible beyond three months, but does suggest a minimum frequency of three remote measurements per week was acceptable to most participants. Further studies should address the clinical implications of integrating remote monitoring of spirometry and pulse oximetry into ILD care and the experience and opinions of ILD clinicians.

## Conclusion

This study demonstrates that remote monitoring of spirometry and oximetry can be integrated into ongoing clinical care of people with ILD. Further research is needed to understand the economic implications of this technology into health services, and the utility of remote monitoring from a clinician’s perspective. We are currently undertaking a multi centre UK randomised controlled trial to investigate this.

### Electronic supplementary material

Below is the link to the electronic supplementary material.

**Supplementary Material 1: Additional Figure 1**: Heatmap demonstrating frequency of spirometry attempts throughout the 91 day study period. Each horizontal bar represents a participant within the study. Each vertical line within a bar represents a day on which home spirometry was performed.

**Supplementary Material 2: Additional Table 1**: Impact of patient factors on desire to continue remote monitoring

## Data Availability

The datasets used and/or analysed during the current study are available from the corresponding author on reasonable request.

## Abbreviations

CHP: Chronic Hypersensitivity Pneumonitis
CTD-ILD: Connective Tissue Disease associated Interstitial Lung Disease
EQ-5D-5L: EuroQoL 5-dimension 5-level questionnaire
FVC: Forced Vital Capacity
HRQoL: Health Related Quality of Life
ILD: Interstitial Lung Disease
ILD-GAP: Interstitial Lung Disease Gender-Age-Physiology Score
IPF: Idiopathic Pulmonary Fibrosis
IQR: Interquartile range
MCID: Minimal clinically important difference
MID: Minimal important difference
NSIP: Non specific interstitial pneumonitis
PAM: Patient Activation Measure
RA-ILD: Rheumatoid Arthritis Associated Interstitial Lung Disease
SD: Standard deviation
TLCO: Transfer factor of the Lung for Carbon Monoxide
VAS: Visual Analogue Score
