# Supplementary figures and images for: Feasibility and acceptability of remotely monitoring spirometry and pulse oximetry as part of interstitial lung disease clinical care: a single arm observational study
Source: Respir Res. 2024 Apr 15;25:162. doi: 10.1186/s12931-024-02787-1 (PMC11020645; doi:10.1186/s12931-024-02787-1)

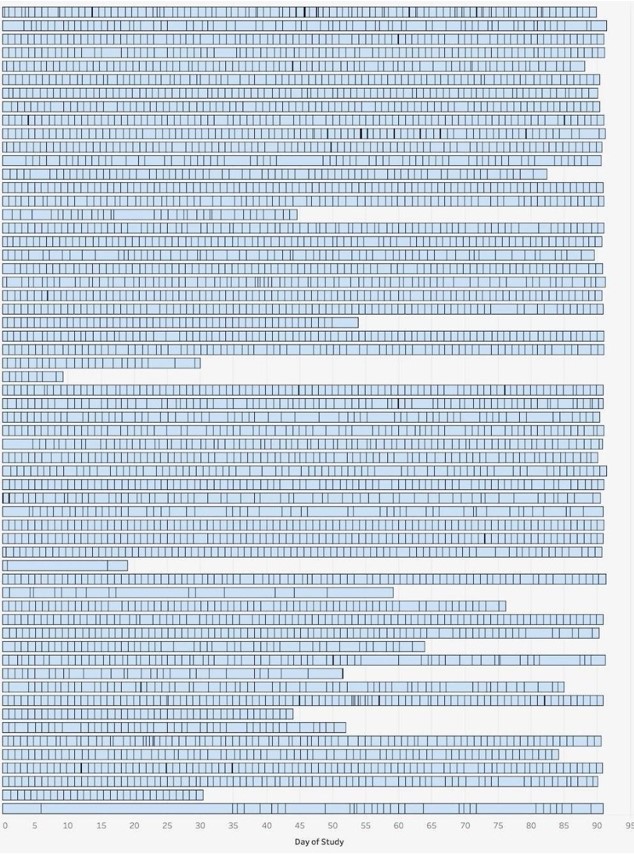

Supplement: Supplementary file 2 — Supplementary Material 2: Additional Table 1: Impact of patient factors on desire to continue remote monitoring [file 12931_2024_2787_MOESM2_ESM.jpg]
