# Supplementary material for: Feasibility and acceptability of remotely monitoring spirometry and pulse oximetry as part of interstitial lung disease clinical care: a single arm observational study
Source: Respir Res. 2024 Apr 15;25:162. doi: 10.1186/s12931-024-02787-1 (PMC11020645; doi:10.1186/s12931-024-02787-1)
Supplement: Supplementary file 3 [file 12931_2024_2787_MOESM1_ESM.odt]

Supplementary Table 1: Impact of patient factors on desire to continue remote monitoring

|  | Participants wished to continue remote monitoring [median(IQR)] | Participants who did not wish to continue remote monitoring [median(IQR)] | p |
| --- | --- | --- | --- |
| n | 24 | 8 |  |
| Age | 71.5 (64.75 – 75) | 69 (66.25-74) | 0.6 |
| PAM level | 3 (3-4) | 4 (3-4) | 0.52 |
| Baseline FVC L % pred | 2.82 (2.29 -3.25) 79.25 (70.75 – 85.6) | 2.97 (2.56-3.92) 79.05 (73.2-96.4) | 0.72 |
| Baseline TLCO (% pred) | 51 (42.9 – 60.12) | 51.3 (39.75-68.45) | 0.84 |
| Gender (n) | 0.82 |
| ILD subtype (n) | 1.0 |

Only p values provided for gender and ILD subtype as small numbers have been suppressed; One participant did not respond to the question about continuing remote monitoring and therefore was not included.
